# Convolutional Neural Networks for Image-Based High-Throughput Plant Phenotyping: A Review

**DOI:** 10.34133/2020/4152816

**Published:** 2020-04-09

**Authors:** Yu Jiang, Changying Li

**Affiliations:** ^1^Horticulture Section, School of Integrative Plant Science, Cornell AgriTech, Cornell University, USA; ^2^School of Electrical and Computer Engineering, College of Engineering, The University of Georgia, USA; ^3^Phenomics and Plant Robotics Center, The University of Georgia, USA

## Abstract

Plant phenotyping has been recognized as a bottleneck for improving the efficiency of breeding programs, understanding plant-environment interactions, and managing agricultural systems. In the past five years, imaging approaches have shown great potential for high-throughput plant phenotyping, resulting in more attention paid to imaging-based plant phenotyping. With this increased amount of image data, it has become urgent to develop robust analytical tools that can extract phenotypic traits accurately and rapidly. The goal of this review is to provide a comprehensive overview of the latest studies using deep convolutional neural networks (CNNs) in plant phenotyping applications. We specifically review the use of various CNN architecture for plant stress evaluation, plant development, and postharvest quality assessment. We systematically organize the studies based on technical developments resulting from imaging classification, object detection, and image segmentation, thereby identifying state-of-the-art solutions for certain phenotyping applications. Finally, we provide several directions for future research in the use of CNN architecture for plant phenotyping purposes.

## 1. Introduction

Food security is one of the biggest challenges for the world. The global population is likely to exceed 9 billion by 2050, which will necessitate more food, fiber, and fuel products from agricultural production systems [1]. To fulfill these increasing demands, current crop productivity needs to be doubled approximately by 2050, which translates into an annual growth of 1.75% of total factor productivity (TFP) [2]. On average, the current TFP annual growth is approximately 1.5% globally, but the TFP annual growth has decreased to 0.96% in developing countries, thus presenting a significant challenge for the improvement of crop productivity. In addition to productivity, sustainability is another crucial factor for agriculture. Crop productivity must be increased in a sustainable way because the global population will continue to increase and could exceed 11 billion by 2100, which will make these situations even more challenging [3]. Agricultural sustainability, however, faces tremendous challenges from decreasing workforce availability, changing climate, shortfall of arable land, and limited water resources [4]. It is thus paramount to improve simultaneously the productivity and sustainability of agricultural production systems.

There are two potential ways to address these issues: improving crops and improving crop management. Improving crops is aimed at breeding new crop cultivars such that crops can naturally have a higher yield, better quality, and improved adaptability to various environments (e.g., saline soils). Improving crop management seeks to advance farming concepts, such as precision agriculture, which minimize the input (e.g., irrigation and chemical application) and maximize the outcome (e.g., productivity and quality) for an agricultural production system through technological innovations (e.g., sensing, automation, and data science techniques). Both ways face the same bottleneck: the evaluation of a large amount of plants in the field. It is therefore paramount to develop new technologies to accurately evaluate crop plants in a high-throughput manner.

**Figure 1:**
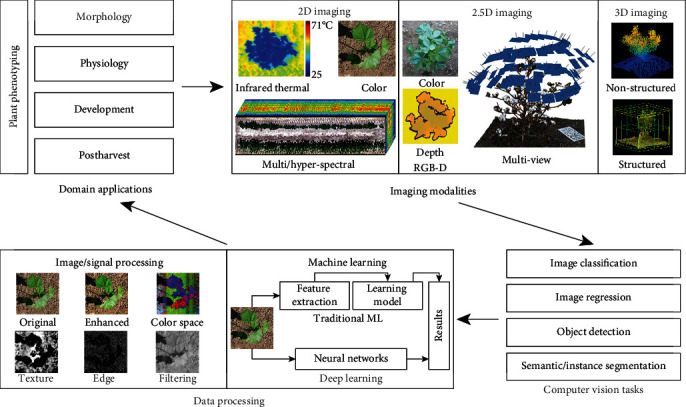
Diagram of the pathway of imaging-based plant phenotyping.

High-throughput plant phenotyping (HTP) has been recognized as integral to overcoming this bottleneck [5–12]. In the past five years, various HTP solutions have been developed to dramatically improve phenotyping capability and throughput, including tower-based systems, gantry-based systems, ground mobile systems, low- and high-altitude aerial systems, and satellite-based systems. An obvious trend has been noticed in the recent HTP systems: imaging sensors have been used more frequently because of their ample capacity for extracting complex traits. 2D imaging (e.g., RGB color, thermal, and spectral imaging) can provide spatial information of a scene plus an additional data dimension such as spectral information from the spectral images. 3D imaging (e.g., LiDAR) can provide a 3D structure of a scene that can be used to calculate object morphological traits (length, area, and volume). 2.5D imaging (e.g., depth camera) retains the structure information of the imaging plane, which is similar to 2D imaging, and acquires the depth information of a scene, which can be used to reconstruct the 3D structure of that scene. Imaging-based solutions have been used for a wide range of phenotyping applications covering plant morphology, physiology, development, and postharvest quality. A typical pathway for imaging-based plant phenotyping can be demonstrated in a four-step cyclic graph (Figure 1). The first step is to identify and define phenotypic traits to be measured, which largely determine the use of suitable imaging modalities for plant sensing. Measuring phenotypic traits usually demands one or more computer vision tasks (e.g., fruit counting may require object detection) that can be solved by developing new or improved algorithms through conventional image/signal processing, machine learning, or a combination of the two. Data processing pipelines can be designed to extract defined phenotypic traits to support and facilitate domain applications such as genetics/genomics studies, breeding programs, and production management. Among these options, algorithm development becomes noticeably challenging because of significant disparities in image quality (e.g., illumination, sharpness, and occlusions) [13]. These image quality variations considerably affect the performance of image/signal processing algorithms and result in poor algorithm generalization for measuring the same phenotypic traits from different datasets. Conventional machine learning- (ML-) based approaches generally have improved generalizability, but most of them still cannot meet the requirement for current phenotypic purposes. In addition, conventional ML approaches require considerable effort to design data representations (features) manually that are invariant to imaging environment changes. Furthermore, feature designing is laborious and requires expertise in computing and image analysis, which prevents the use of conventional ML techniques for phenotyping applications.

Deep learning (DL) is a subset of machine learning and allows for hierarchical data learning. The key DL advantage is that features will be learned automatically from input data, thereby breaking down barriers to the development of intelligent solutions to different applications. A commonly used DL architecture is deep convolutional neural networks (CNNs), which have achieved state-of-the-art performance for important computer vision tasks, such as image classification/regression, object recognition, and image segmentation (both semantic and instance). CNNs originated in the1980s [14] and showed their first success in the recognition of handwritten digits in the 1990s by using backpropagation-based training [15]. In 2012, a breakthrough (AlexNet) was made because of significant improvements in computational power (and therefore CNN model complexity) and the availability of annotated datasets (e.g., ImageNet) [16]. Since then, various types of CNN architecture have been developed for image classification and eventually have demonstrated better performance than humans on the same dataset [17]. In addition, CNNs have been used widely as feature extractors integrated with meta-architecture for other computer vision tasks, such as object detection and semantic and instance segmentation. CNNs have provided state-of-the-art performance in comparison to traditional approaches for almost all of these tasks, demonstrating great potential for the improvement of data analysis performance in such imaging-based applications as imaging-based plant phenotyping. In particular, the advancement of transfer learning (a technique that helps to transfer features learned from one dataset to another, benefiting applications with limited annotated data from large publicly available datasets) and the emergence of DL libraries further facilitate the use of DL techniques for domain applications, so DL approaches have been adopted rapidly for plant phenotyping in recent years, and an exponentially increasing trend is foreseen for DL-based plant phenotyping. It is thus necessary to conduct a literature review to summarize the existing knowledge, good practices, limitations, and potential solutions to applying DL techniques in plant phenotyping.

Several papers have been published in the last two years that provide comprehensive reviews of DL techniques for such computer vision tasks as image classification [18, 19], object detection [20], and semantic segmentation [21]. These reviews effectively summarize the basic principles, development history, and future trends for CNNs in computer vision, but none of them provide information related to agriculture, which highlights a gap between these technological theories and phenotyping applications. There have been pioneering efforts that have focused on various DL techniques for general agriculture applications [22] and plant stress phenotyping [23]. They were, however, either too broad (covering all DL techniques for all agricultural applications) or too narrow (limited to a particular phenotyping task) and lack a focused and comprehensive review of DL in imaging-based plant phenotyping.

The goal of this review is to scrutinize thoroughly the current efforts, provide insights, and identify potential research directions for the utilization of CNNs for imaging-based plant phenotyping. This review focuses on key phenotyping tasks related to plant stress, development, and postharvest quality. By addressing this gap in research, it is expected that readers can bring CNNs into their research to benefit the plant phenotyping community. Deep learning and plant phenotyping are emerging research fields and grow extremely rapidly, so this review primarily focuses on studies published (1) in peer-reviewed (or open-reviewed) journals and conferences; (2) in the most recent 5 years (2015 to 2020); and (3) in the use of CNNs for imaging-based plant phenotyping. Literature has been collected from three main resources including Elsevier ScienceDirect, IEEE Xplore Digital Library, and Google Scholar. Keywords of “CNN” and phenotyping tasks (i.e., “plant stress”, “plant development”, “fruit counting”, “flower counting”, “root phenotyping”, and “postharvest quality”) were used as combinations for literature searching. The rest of this review is organized in the following way: Section 2 provides a concise introduction to important CNN architecture used in image classification, object detection, and semantic and instance segmentation; Section 3 provides a review of CNNs for image-based plant phenotyping; Section 4 discusses key issues in using CNNs for plant phenotyping; and Section 5 provides conclusions and potential directions for future research.

## 2. CNNs for Computer Vision Tasks

Since 2012, CNNs have dominated the solutions for computer vision tasks because of their superior performance. While efforts have been made to review thoroughly the development of various CNN architecture for computer vision tasks [18–21], we have provided a brief introduction to make this review more comprehensive. Most imaging-based phenotyping applications essentially demand solutions for one or more tasks related to image classification, object detection, and segmentation, so CNNs for those tasks are reviewed in this section. Because CNNs evolve rapidly, the following review is limited to models that provide significant performance improvements and are used widely as benchmark methods by other domain applications. For convenience, useful information is summarized for those reviewed models, including development year and group, the original reference, the key innovation concept, and the source code (or third-party implementation) if available (Table 1).

### 2.1. Image Classification

**Figure 2:**
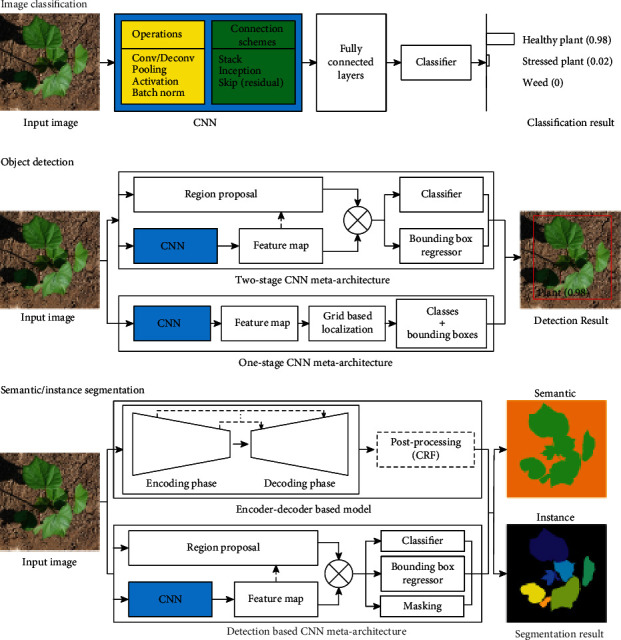
Diagrams of CNN architecture mechanisms for image classification, object detection, and semantic and instance segmentation.

Image classification is one of the core tasks in computer vision and is aimed at assigning images with predefined class labels. CNNs are artificial neural networks that combine a set of mathematical operations (e.g., convolution, pooling, and activation) using various connection schemes (plain stacking, inception, and residual connection), and the operational parameters (e.g., convolutional kernels) can be learned from annotated images to predict image class labels (image classification in Figure 2). The development of modern CNNs for image classification can be grouped into three stages: (1) emergence of modern CNNs (2012 to 2014); (2) intensive development and improvement of CNN architecture (2014 to 2017); and (3) reinforcement learning for CNN architectural design (i.e., the concept of using artificial intelligence (AI) to improve AI, 2017 to present).

In 2012, the first modern CNN architecture (also known as AlexNet) was reported and demonstrated breakthrough performance on image classification in the 2012 ImageNet Large Scale Visual Recognition Challenge (ILSVRC 2012) competition [16]. It showed improvements of 8.2% and 8.7% on top-1 (35.7% versus 45.7%) and top-5 (17% versus 25.7%) errors. This work began the new round of using CNNs for image classification and other computer vision tasks. Researchers intensively studied CNN architecture for imaging classification from 2014 to 2017 and developed several representative CNNs such as VGGNet [24], Inception-based CNNs [25], ResNet and its variants [17], and DenseNet [26]. These CNNs showed dramatical improvements of learning capability and computational complexity through the use of efficient operations (e.g., a 3 by 3 convolutional operation as the building block) and revised connection schemes (e.g., inception modules, residual modules, and dense blocks). With these improvements, representative CNNs can now usually surpass human performance on image classification for various datasets. It should be noted that performance improvement following CNN architectural modification was heavily dependent upon human expertise and tuning efforts, which means that CNN architectural improvement could be as laborious as feature engineering in traditional ML. To solve this problem, a study was conducted to explore the possibility of searching optimal CNN architecture using reinforcement learning, which is a learning method to reward operations yielding improved performance [27]. A reinforcement learning framework was introduced to seek optimal convolutional cells on a small annotated dataset, and the resultant cells were stacked in different ways and transferred to a large unknown dataset. Experimental results showed that CNNs built by searched convolutional cells provided varying degrees of performance improvement over CNNs designed manually. This demonstrates the capability of using AI to improve AI, which is a new direction for solving some of the problems associated with designing CNN architecture. The search process, however, is extremely expensive computationally (500 NVIDIA P100 GPUs for 4 days), which limits its potential use for other domain applications.

In addition to performance improvement, studies have been conducted to understand the mechanism of CNNs. This leads the development of techniques towards explainable artificial intelligence which helps develop interpretable and inclusive machine learning models and deploy the models with confidence. A pioneering work improved AlexNet to a new variant (ZFNet) using a visualization tool. This visualization tool is a framework integrated with CNNs that can map neuron activities back to the input pixel space. Pixel-wise activations, therefore, can be visualized after each convolutional layer. This would be particularly useful for researchers seeking to understand the CNN mechanism and improve architectural design. The study also showed that learned features could be generalized to various classifiers, suggesting CNNs could learn general representations of images rather than specific features for classification. Successive studies furthered this direction and developed various gradient-based methods that can visualize the importance/relevance of features to classification results. Commonly used methods include guided backpropagation, gradient-weighted class activation mapping (Grad-CAM), and layer-wise relevance propagation (LRP). Some general framework (e.g., LIME and occlusion map) can also be used to reveal important image regions to classification results. Details of these visualization methods can be obtained in separate reviews [28, 29].

### 2.2. Object Detection

Object detection seeks to detect and classify all potential objects in a given image. The use of CNNs for object detection can be categorized into two groups: one-stage and two-stage CNN architecture (object detection in Figure 2). Two-stage models firstly detect candidate object regions (region proposal) and subsequently classify the candidate regions into different object categories (region classification). Intuitively, existing region proposal methods can be combined with CNNs as two-stage models for object detection. The OverFeat framework was developed to use a single CNN to extract features for training classifiers and regressors separately [30]. The trained classifiers and regressors were used to predict class labels and bounding box coordinates, respectively, for candidate ROIs generated using a sliding window method. Although the OverFeat framework provided the best performance on the localization task of the 2013 ILSVRC competition, the high computational cost and training complexity presented difficulties for practical applications. A region-based CNN (RCNN) family was introduced to resolve those issues, including the original RCNN [31], Fast RCNN [32], and Faster RCNN [33].

Three key techniques were identified in CNN architecture of the RCNN family, including the region proposal network (RPN), ROI pooling operation, and multitask loss function. An RPN was developed to generate candidate object ROIs using features extracted from CNNs, which simultaneously saved processing time and increased region proposal accuracy. An ROI pooling operation was developed to extract a fixed number of features from ROIs with varying sizes, thereby avoiding the repeated computation of features for different ROIs. A multitask loss function was used to unify the training process, which enabled an end-to-end training for object detection. With these three improvements, Faster RCNN has been used widely as either a benchmark for performance comparison or an object detector for domain applications (e.g., pedestrian detection in autonomous driving) because it is easy to train and generally provides accurate detection performance. Although Faster RCNN provides state-of-the-art accuracy, its efficiency is still inadequate for use in real-time applications such as autonomous driving. This is mainly because the two-stage models spend time handling different components for inference [20]. Compared with two-stage models, one-stage models can reduce time expense by global regression/classification by mapping directly from image pixels to bounding box coordinates and class probabilities. In other words, candidate object regions are generated from each pixel in feature maps and then classified and fine-tuned to create accurate object boundaries.

Representative one-stage models include the you-only-look-once (YOLO) family [34] and the single-shot detector (SSD) framework [35]. A critical issue, however, has been discovered for these one-stage models: an extreme imbalance in the number of object and background regions. Most image regions contain only the background information (identified as irrelevant regions), providing a limited contribution to the model training process. A focal loss function has been proposed to further penalize inaccurately detected (or classified) samples, which solves the issues resulting from sample imbalance and ultimately leads to the development of an improved one-stage framework RetinaNet [36]. When using the same CNN backbone model, RetinaNet achieved comparable performance with Faster RCNN and 29% improvement of computational efficiency. Nevertheless, if detection accuracy is the most important factor to be considered, two-stage models would be the option; otherwise, one-stage models provide better computational efficiencies for embedded systems and real-time applications.

### 2.3. Semantic and Instance Segmentation

Semantic segmentation seeks to provide masks for objects with the same semantic meaning (e.g., all plants in an image), whereas instance segmentation seeks to provide individual objects in a given image. In general, CNN architecture for semantic/instance segmentation can be classified into two groups: encoder-decoder-based frameworks and detection-based frameworks (semantic and instance segmentation in Figure 2).

Encoder-decoder-based models usually contain two phases. The encoder phase uses CNNs to extract feature maps that are semantically meaningful from input images, and the decoder phase uses transposed convolution (also known as deconvolution) for upsampling of extracted feature maps to per-pixel labels. Two techniques have been used to improve the segmentation accuracy of encoder-decoder models. First, a lateral connection scheme is used to link feature maps with the same spatial resolution between the encoder and decoder phases, which aids in the preservation of semantic meaning from input images to output segmentation results [37]. Second, a conditional random field (CRF) is used as a postprocessing method to improve the segmentation accuracy of object boundaries [38]. Representative encoder-decoder-based models include U-Net [37], fully convolutional network (FCN) [39], and DeepLab [38]. A detection-based framework relies on CNN architecture for object detection. Several studies have explored the use of object detection models for instance segmentation, including simultaneous detection and segmentation (SDS) based on RCNN [40] and DeepMask based on Faster RCNN. They did not, however, reach an acceptable performance for the instance segmentation task [41]. A breakthrough performance was achieved by Mask RCNN that supplements an FCN network with a Faster RCNN for generating masks of individual objects [42]. Many later studies and applications have also proven that the Mask RCNN could provide state-of-the-art performance for semantic and instance segmentation.

## 3. CNN-Based Analytical Approaches for Image-based Plant Phenotyping

### 3.1. Plant Stress Phenotyping

Plant stress phenotyping is aimed at identifying and evaluating plant responses to abiotic and biotic stresses, providing information for the selection of accession lines with high stress resistance and tolerance in breeding programs and the understanding of intrinsic mechanisms in genetics/genomics studies. In addition, plant stress detection, especially in early stages, is crucial for data-driven pest and weed management in agricultural production systems. Plant stress phenotyping can be categorized into four stages: (1) identification (presence of stress); (2) classification (type of stress); (3) quantification (severity of stress); and (4) prediction (possibility of stress occurrence) [23]. From the computer vision perspective, all four stages can be considered an image classification task, whereas some stages could involve other processing methods, such as object detection and semantic/instance segmentation.

The development of image classification-based approaches can be divided into two phases. In the first phase, studies intensively investigated well-known and custom CNN architecture because of the availability of annotated datasets and the simplicity of CNN implementation and training for image classification. Several large, annotated image datasets for plant stress classification accelerated the evaluation of various CNNs for stress phenotyping. For instance, PlantVillage (https://plantvillage.psu.edu/) is a publicly available image dataset containing over 54,000 labeled plant leaf images from 14 crop species with 26 types of stress. This can be used to either evaluate a new CNN architecture or pretrain a CNN model for transfer learning. Data annotation for image classification is also relatively easy (compared with object detection and semantic/instance segmentation), so a large number of images in a newly collected dataset can be annotated within a reasonable time and cost, especially when a proper data collection procedure is used. As a result, studies related to plant stress detection typically have a sufficient number of annotated images (several thousand or more) for model training. In addition, DL libraries have been developed to accelerate the implementation and training of CNNs for image classification. Commonly used DL libraries include Caffe (University of California Berkeley), Theno (University of Montreal), TensorFlow (Google), PyTorch (Facebook), CNTK (Microsoft), and Keras (open source). Key CNNs (e.g., Inception-based CNNs, ResNet family, and DenseNet) have been implemented using various libraries, so researchers can develop computer programs quickly for training CNNs provided the annotated data are available. These advancements facilitate the use of CNNs for plant stress identification at the image level. By using good training practices (e.g., data augmentation, background removal, and transfer learning), various studies have shown that CNNs achieved accuracies from 87% to 99% for stress identification and classification [43–54]. Details of these studies can be accessed in a latest review [23].

**Figure 3:**
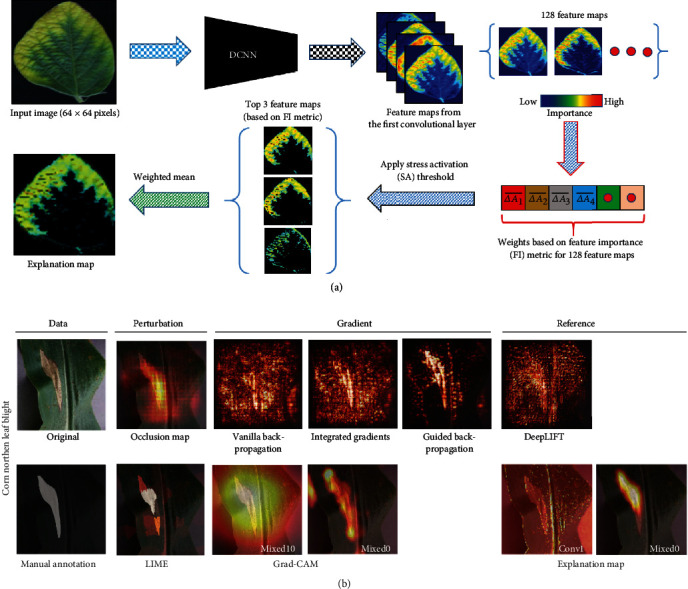
Key concept and results of xPlNet for plant stress detection: (a) diagram of the developed xPlNet for calculating the explanation map for a given image; (b) visualization results using different methods for an image containing a stressed leaf. (a) and (b) were reproduced using figures from [55, 56], respectively.

In the second phase, pioneering studies attempted to understand reasons leading to high performance of CNNs for stress identification and classification, because the understanding would not only help to improve CNNs but also ensure biological correctness of obtained results. Although some studies adopted the deconvolution layers to visualize the activated pixels in different convolutional layers, the visualization results were not used to compare with human evaluation or correlate with biological knowledge. Through 2018, an explainable framework (xPlNet) was in development that could both identify (or classify) plant stresses and generate an explainable map showing pixels that determined identification (or classification) results (Figure 3(a)) [55]. In this framework, the reference activation level (the mean pixel intensity plus 3 times the pixel intensity variation) of healthy leaves was calculated for each of the feature maps extracted in the first convolutional layer. For a testing image, feature maps from the first convolutional layer subtracted the reference activation to calculate the feature importance metric (weighted average of leaf pixel intensity in each of feature maps). Feature maps were ranked based on their importance, and the top-*K* (*K* = 3 in the original study) feature maps were selected to calculate the explainable map (EM). The mean intensity of the EM can be used to quantify stress severity. A separate study also examined various techniques to understand the mechanism of CNNs for disease diagnosis [56]. Explanation maps generated by xPlNet generally showed the best correlation with manual annotation and validated its efficacy for finding pixels correlated to stressed lesions (Figure 3(b)). Compared with studies in the first phase, the two pioneering studies demonstrated the importance of understanding the mechanism of CNNs for stress phenotyping as well as the potential for stress severity quantification. Image annotation is still recognized as a limiting factor for using many DL algorithms (especially supervised ones), so researchers investigated the use of generative adversarial networks (GANs) to generate synthetic images for training CNN models for plant stress detection and classification [54]. AR-GAN based on Cycle-GAN was developed to translate contextual information learned between different image sets. For instance, lesions in infected leaf images can be transferred to healthy leaf images or vice versa. With that, one can expect to substantially increase the number and diversity of images for model training.

In addition to image classification-based approaches, improved CNN models for object detection were used for plant stress phenotyping [46]. Three representative architecture (Faster RCNN, SSD, and R-FCN) were trained and evaluated, and experimental results showed that the best detection accuracy was 86% at an intersection over union (IOU) level of 0.5. IOU is defined as the intersection area between two objects over their union area, which can be used to evaluate the object overlap: 0 for no overlap and 1 for perfect overlap. Trained architecture could identify and localize the symptomatic regions. Since plants can be infected by multiple diseases, the object detection-based solutions could detect all possible causes, thus providing a more comprehensive evaluation than image classification-based solutions. A study was conducted to generate a heat map of stressed lesion probabilities from small patches obtained by using a sliding window over a given image [45]. The generated heat maps were used as input images for a separately trained CNN for detection of stressed lesions. The developed method showed two advantages. First, high-resolution images were processed directly without downsampling, so detailed spatial information could be utilized by CNNs. Second, the generated heat maps were used as a visualization tool to explain classification decisions. This advantage, however, was not recognized and fully explored. In addition, generated probability maps can be used to segment stressed lesions by using postprocessing methods such as conditional random field (CRF) [57]. Plant stresses can be then quantified easily using the ratio of stressed pixels to healthy pixels, which provides a quantitative metric for stress severity evaluation. Semantic and instance segmentation could be more straightforward approaches to obtain masks of stressed lesions in images. This could be an important direction for future research, although pixel-level annotation can be extremely costly.

Advanced imaging modalities (e.g., hyperspectral imaging) capture plant data in a wider spectrum than RGB imaging, providing useful information for plant stress identification. A very recent study explored the use of a custom CNN architecture to detect plant diseases in hyperspectral images [58]. The novelty of the custom architecture is the use of a 3D convolutional operation that can directly convolute both spatial and spectral information in hypercubes. This would not only inspire future studies related to plant stresses but also enable the reanalysis of many previous hyperspectral images collected for plant stress analysis. With an improved detection accuracy, subtle stress differences among cultivars/treatments may be revealed to enhance our understanding of plant responses to stresses.

### 3.2. Plant Development

#### 3.2.1. Plant Shoot Morphology and Growth

Morphological changes of plant shoot are key to describing plant development. Canopy coverage and leaf area are two commonly used parameters to quantify plant growth and development, especially in aerial image analysis. Calculating the two parameters requires accurate plant segmentation. Many studies have used color-based features (e.g., excess green index) to segment plants, but they usually had imperfect segmentation because plant color could have large image-by-image variations due to illumination, shadowing, occlusion, and so on. Therefore, some studies explored the use of CNNs for plant segmentation [59–64]. Most of them considered plant segmentation a semantic segmentation task and used encoder-decoder-based CNN architecture for processing. Although these studies demonstrated improved segmentation accuracy, training data annotation for semantic segmentation can be extremely laborious. To solve this issue, a study attempted to generate synthetic images along with semantic annotations automatically for CNN model training [64]. Combining synthetic and real images would improve the generalizability of CNNs for plant segmentation and thus growth analysis accuracy.

Two studies treated morphological measurement as an object detection problem [61, 65]. The first study used a Faster RCNN to detect citrus trees and obtain tree image patches, so tree canopies could be easily and accurately segmented in individual image patches by using a thresholding-based method [61]. The second study, however, attempted to detect key points (e.g., ground-plant junction point and topmost point of main trunk) of plants/plant leaves, so morphological traits (e.g., plant height and leaf length) were measured based on the exact biological definitions [65]. Compared with traditional computer vision methods, this CNN-based solution could measure a morphological trait in a way closer to its biological definition. For instance, plant height is defined as the distance from the ground to the topmost main stem point for most crops. Many studies, however, used an approximate measurement which is the distance from the ground to the topmost canopy point because of the difficulty of finding the topmost main stem point (even the point presents in images). By using the CNN-based solution, one can expect to get more accurate morphological measurements and may have more possibility to resolve subtle differences among plants.

Researchers also combined CNNs with other DL methods (e.g., recurrent neural network (RNN)) for plant development characterization [66, 67]. CNNs were used as a feature extractor to encode plant spatial status in individual growth stages, and RNNs (e.g., long-short-term memory (LSTM)) were used to embed all spatial encodes to learn plant temporal changes. In this way, plant growth patterns could be fully encoded by neural networks to reveal differences among crop cultivars and treatment groups. This indirect phenotyping scheme could be particularly useful for selection-oriented programs, but explaining the selection would be a significant challenge and barrier for many research studies that aim to understand the mechanism of many plant responses. Thus, it is important to further develop visualization tools to enhance the explainability and interpretability of complex neural network architecture.

In addition to morphological measurements, CNNs could be used to monitor certain plant development events such as plant lodging [68]. A new CNN architecture (LodgeNet) was developed by integrating a custom 7-layer CNN model with handcrafted features (i.e., local binary pattern and gray-level cooccurrence matrix). Compared with 10 well-established CNN architecture, LodgeNet provided comparable or better performance on the differentiation between lodging and regular plots but with a considerable improvement in processing speed (at least 2 times faster). It is noteworthy that transfer learning in this study was not as efficient as other studies because of the use of multispectral images, which might limit the capability of those well-established architecture. Nonetheless, this study demonstrated the potential of combining a shallow CNN with handcrafted features for fast training and inference, which can be very useful for applications that require real-time processing or have limited computing resources.

#### 3.2.2. Plant and Plant Organ Counting

Counting plants and plant organs are central to characterizing plant development. This section provides a comprehensive overview of studies related to the detection and counting of plants and plant organs. Based on data format, these studies can be classified into two categories: (1) detection and counting in still images and (2) detection and counting in image sequences and videos.

**Figure 4:**
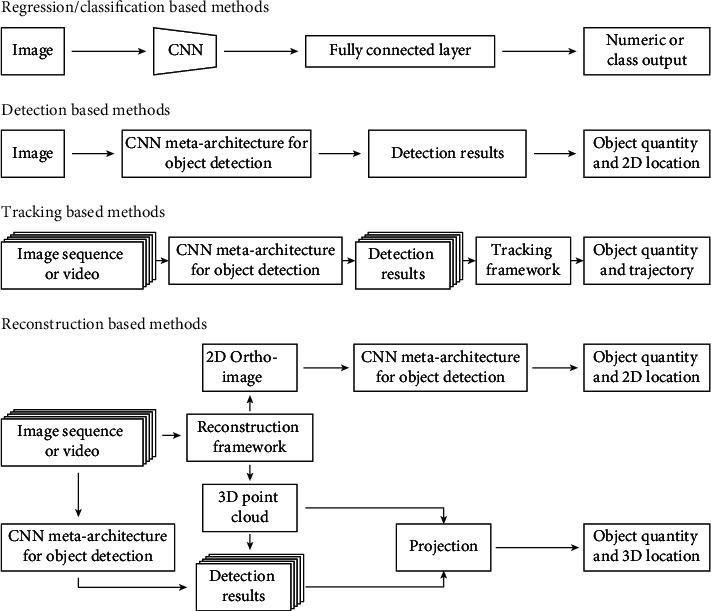
Diagrams of key concepts for using CNNs for plant/organ detection, counting, and localization.

*(1) Counting in Still Images*. Regression or image classification (can be considered discrete regression) is the simplest and most straightforward way to fruit/organ counting from the technical development viewpoint (regression-/classification-based methods in Figure 4). For regression-based methods, a major modification is made that replaced the softmax layer of a CNN with a single neuron for regressing numeric values (e.g., fruit counts). This simple end-to-end counting solution provided high accuracy (over 90%) for counting fruits and plant leaves [69–80]. In particular, an Arabidopsis dataset with finely grained annotations has been developed to open opportunities for the development of advanced analysis methods [81]. One of the dataset's tasks is leaf counting. Many studies have been reported in workshops on the Computer Vision Problems in Plant Phenotyping (CVPPP) for the leaf-counting problem [69–71, 77, 78].

A particular challenge of the regression-based solutions is the limited availability of annotated images, leading to many potential concerns such as poor model generalizability. To solve this issue, a study attempted to generate synthetic data of tomatoes to enhance the data availability and diversity [69]. Green and brown circles with different sizes were used to fill the entire image as background, and red circles with different sizes were rendered on top of the background to simulate tomatoes. Although trained CNNs achieved a counting accuracy of 91% on real images, the study only tested red tomatoes, which have distinctive color features from the background. The generalizability of this approach should be further validated for challenging situations such as detection of green tomatoes from leaves. GANs were also used to generate synthetic data for model training [71]. Compared with the method used in [69], GANs could output images with realistic texture and structure. This would help to address the potential generalizability issue due to image variations. An alternative approach was to use patch-based training. TasselNet was developed to count maize tassels in two steps [72]. In the first step, a local CNN regression model was established to predict the number of tassels in each patch of an image. In the second step, the estimated count in each image patch was averaged based on individual pixels in that patch to create a counting map with the same spatial size as the original image. The sum of all pixel intensities in the counting map represents the final tassel count in that image. Experimental results showed that TasselNet achieved counting accuracies from 74.8% to 97.1%, which were 2 to 5 times higher than with conventional methods. TasselNet uses the patch-based training method, which substantially increases the number of images for training. In addition, TasselNet requires dot annotation rather than bounding boxes, which further reduced the difficulties in image annotation. A successive study further modified TasselNet by expanding its receptive field to encode context information and a global regression model for count estimation, leading to improvements of both count accuracy and processing speed [79]. In addition to data annotation, studies investigated the use of nonsupervised (e.g., weakly supervised and unsupervised) domain adaption to improve the model generalizability to unseen datasets [76, 78, 80]. Adversarial modules were used to either simplify the annotation from exact object counts to object presence [76, 80] or fine-tune pretrained CNN layers to encode unseen images in a similar distribution to pretraining images for counting regression [78]. A very recent study also reported the use of visualization tools to explain CNN-based counting regression models [77]. Experiments showed that plant leaf boundaries were the most informative parts rather than leaf petioles and centers for leaf counting. In addition, CNNs would encode some image information irrelevant to leaf counting, which could be used to guide neuron pruning to increase the computational efficiency. This demonstrated a promising way of using visualization tools for CNN improvement, explanation, and interpretation. A common finding has been identified in all these studies: a moderately complex CNN is recommended because of the potential of model overfitting. This could be of particular concern for regression-based counting methods, as its learning target is much simpler than either image classification or object detection. Another noteworthy drawback is that no location information can be provided by regression-based methods, which limits the potential for using these methods for other applications. For classification-based methods, plant/organ counting was treated as a discrete counting (or scoring/grading) problem and, thus, a predefined score or grade (e.g., 10% of inflorescence) was assigned to a given image rather than an exact count [82].

An example of the classification-based method is WheatNet, which was developed to predict the percentage of flowering in wheat images [82]. Multiple images were acquired for each plot. A total of 11 classes were annotated for each plot (and thus images for that plot), corresponding to 11 visual scores with a percentage heading from 0 to 100% with an interval of 10%. The average prediction of all images in a plot was the final percentage heading for that plot, which reduced counting errors because of inaccurate classification. By fitting the per-plot percentage heading into a sigmoid function, an error of 1.25 days was achieved between the manual and CNN-based measurements of the heading date (50% of emerged heads), which resulted in the high accuracy of the developed method. It should be noted that heading dates estimated using WheatNet counts showed comparable broad sense heritability (*H*^2^ = 0.987) to those estimated using manual counts (*H*^2^ = 0.982), indicating a great potential for incorporating DL for plant phenotyping and, therefore, breeding programs and genetics/genomics studies. However, the developed method has the major limitation of potential difficulty in generalizing the method for other plants with complex canopy structures and flowering patterns, such as cotton. Flowers in those plants are usually inside canopies instead of on the top of canopies, which can increase partial or full occlusions. If flowers cannot be imaged, it is not feasible to train any ML/DL model for detection and counting. Researchers also used the classification-based method for counting the number of pods in soybeans and obtained a similar performance as human experts [83]. However, classification-based counting methods have the same issue as regression-based methods, which cannot provide the necessary location information for understanding plant development.

Object detection is an intuitive approach to count plant and plant organs in still images: accurate object detection ensures accurate object counting (detection-based methods in Figure 4). DeepFruits was the first study to explore the use of modern CNN architecture (i.e., Faster RCNN) for fruit detection [84]. Several key contributions were recognized in this study. First, transfer learning was used to train a Faster RCNN model with 100 labeled images, demonstrating the potential of using limited labeled images to train CNN architecture. Second, when using RGB images, the trained Faster RCNN model provided a 1% improvement of the F1 score over that of the CRF model. Third, data fusion was conducted at the raw-data level and decision level for Faster RCNN models. Experimental results showed that decision-level fusion further improved the F1 score to 0.838 (an additional 2% compared with Faster RCNN without fusion). However, raw-data level fusion showed a 2% reduction of the F1 score compared to that of the RGB-based Faster RCNN. There were two possible reasons for this reduction. First, the decision-level fusion contained two Faster RCNN models, which had twice the parameters that a single Faster RCNN model had to model image data distribution, which ultimately resulted in the performance improvement. Second, the performance reduction of raw-data level fusion resulted from the pretrained weights on the ImageNet dataset being more suitable for RGB color images than NIR images.

Although these two explanations are reasonable, a more plausible reason might be the ineffective transfer learning of the revised Faster RCNN architecture. In order to use four-channel (RGB-NIR) images for training, the receptive field of the first layer in the backbone CNN was changed from 3 to 4, meaning each filter in the first layer had an additional dimension that had to be initialized randomly. As a consequence, the output from the revised first layer was not likely to follow the data distribution pretrained on the ImageNet dataset, and this new data distribution could eventually corrupt the rest of the CNN because CNNs are hierarchical and deep layers are dependent upon shallow layers [18, 19]. In other words, pretrained weights in deep layers could not effectively model data, which would result in lower transfer learning efficiency. Even worse, if pretrained weights are somehow in a local minimum or saddle, transfer learning might provide worse results than randomly initialized weights. Finally, the study also applied the Faster RCNN model for other fruits such as cantaloupes, apples, avocados, mangos, and oranges, which demonstrated the generalizability of Faster RCNN for fruit detection. While the study generated much useful data, a major drawback was the limited images for training and testing. Although 100 images could let researchers train a Faster RCNN with a higher accuracy, the testing image sizes (from 11 to 34) were too small to confirm the achieved high performance. In particular, training and testing images were acquired in the same condition, which significantly reduced the variation of images. This may also be one major reason that CNN-based solutions showed only marginal improvements over conventional methods.

Many studies have generally followed similar practices and used the region-based CNNs (e.g., RCNN and Faster RCNN) for plant/plant organ counting [85–91]. Two critical issues, however, were not addressed by these studies. The first issue relates to model training. High-resolution images are typically large and cannot be fed into a CNN model for training. A new approach was developed to solve this issue by splitting one high-resolution image into multiple small patches. Each patch still had a relatively large size (e.g., 500 by 500 pixels), so all of the patches could be used to train complex CNN architecture such as Faster RCNN with high-resolution images [92]. In the testing stage, an image was split into patches with a certain overlap (e.g., 50% between two neighboring patches) and a Faster RCNN model was used to detect maize ears in each patch. Because of the considerable overlapping among the patches, one ear could be detected in multiple patches. Overlap between each pair of detections was calculated to remove repeated detections. This strategy substantially increased training samples and was able to process images with an arbitrary resolution. The second issue involved the detection of small-sized objects, which is also a common challenge for CNN-based object detection methods [20]. An intuitive solution was to use features from shallow layers for regional proposals because features from shallow layers reserved more spatial information and could identify small-sized objects. Based on this, features from multiple layers (shallow, middle, and deep) were used for regional proposals of the Faster RCNN models [93]. Compared to standard Faster RCNN models, the modified Faster RCNN model improved the F1 score by 4.7% for detecting almonds in still images.

Apart from Faster RCNN, a custom two-stage framework has been proposed that uses superpixels generated by the simple linear iterative clustering (SLIC) algorithm as region proposals [94]. A CNN model was used to classify each superpixel as either a flower or a nonflower object. While this approach showed higher performance than conventional ML methods (e.g., color features and SVM classifier), it has a potential limitation in region proposal. The advantage of end-to-end CNN architecture is that they are able to use richer features for accurate localization, especially when images vary dramatically. However, superpixels are subject to image variation and might not provide optimal region proposals. The generalizability of this approach, therefore, is very likely to be inferior to that of the end-to-end methods.

In addition to the two-stage architecture, one-stage models have been investigated for situations requiring fast processing. YOLO-v2, for instance, has been used to detect and count apples and pears in still images [95]. Compared with the original YOLO-v2 model, a modification was made to increase grid cells from 13 by 13 to 26 by 26 so relatively small apples could be detected. The modified YOLO-v2 model achieved an F1 score of 0.9 at the IOU level of 0.5. Because the study was concerned with inference speed, the authors halved the YOLO-v2 model from 24 layers to 12 layers, thereby providing a dramatic increase of processing speed (from 4 FPS to 10 FPS) with an acceptable accuracy reduction (F1 score from 0.9 to 0.8). This study also used a rule-based method to generate synthetic images to increase the training data size and diversity, which led to an improved detection performance.

Many studies also investigated semantic segmentation-based approaches to plant/plant organ counting [96–101]. CNN architecture for semantic segmentation were firstly used to obtain plant/plant organ masks. Subsequently, the obtained masks were postprocessed using conventional computer vision methods (e.g., circle fitting and connected component labeling) to isolate individual plant/plant organs, so objects could be counted. A noticeable concern is that although CNNs could provide accurate semantic masks, the counting accuracy can still suffer from inaccurate postprocesses. To address this concern, studies explored the use of instance segmentation CNNs (e.g., Mask RCNN) that can directly segment individual objects in images [102–106]. These studies faced the same challenge in the lack of training data. Training these models usually requires a large number of images with pixel-level annotation, but data annotation at the pixel level is considerably costly and becomes a major limiting factor for applications. To overcome this limitation, most of these studies developed algorithms to generate synthetic images for model training. Two types of image synthetization methods were proposed: rule-based and GAN-based. Rule-based methods use a predesigned leaf model to generate a plant based on predefined plant growth rules (e.g., L-system for Arabidopsis) [102, 105]. During plant image generation, although leaf size, angle, and color could be adjusted, generated plants still lacked textural information on the leaf surface, which might lead to a poor performance of trained models. GAN-based approaches, however, could generate synthetic images without the sacrifice of leaf texture. Thus, a method was developed by combining rule-based methods and GANs for image synthetization [103]. The method consists of a rule-based generator for plant mask image and a conditional GAN (c-GAN) for plant color image. A plant mask image is firstly generated based on the predefined leaf model and growth rules and then fed into the c-GAN to map the plant mask to an artificial color image of that plant. By combining the real and synthetic datasets for training, trained models achieved the best performance in both leaf instance segmentation and counting. The developed hybrid method for image synthetization could be potentially used for other applications with limited annotation data. On the other hand, training the c-GAN (or general GANs) is not trivial and requires extensive experiences in model tuning, which can be the barrier for domain experts (e.g., biologists) to directly adopt the method.

*(2) Counting in Image Sequences and Videos*. Although the aforementioned studies have demonstrated that the detection and counting of plants and plant organs can be fairly accurate in still images, a single image is usually not adequate to cover a plant of tree crops (e.g., an apple tree) or an entire plot of row crops. Thus, image sequences and videos need to be acquired, and processing these data requires expanding detection and counting methods. The key challenge of object detection in image sequences or videos is to associate the same object over different images. There are currently two types of methods that address this issue: tracking-based methods and reconstruction-based methods.

The key to tracking-based methods is to associate detections of the same object (correspondence estimation) over consecutive image sequences or video frames so that individual objects can be tracked to avoid repeated counts (tracking-based methods in Figure 4). With regard to correspondence estimation, there are two methods. The first type is based on trajectory information, which can be acquired using sensors such as RTK GPS and IMU devices. For instance, a framework has been developed to count mangos for yield estimation [107]. This framework firstly detected mangos in each still image using a Faster RCNN model. Camera location and pose parameters were collected for each image so that the geometric correspondence could be calculated between pixels in two consecutive images. Thus, it was able to associate and track mango detections from one image to the next. Experimental results showed that the developed framework achieved an accuracy of 98.6% for mango counting with an inexpensive computational cost, thus demonstrating the efficacy and efficiency of tracking-based methods. The developed framework had three limitations, however. First, the use of positioning devices would increase the cost of the data acquisition system, which could be an issue for small farms and research projects that lack adequate funds. Second, the accuracy of geometric correspondence is dependent upon the accuracy of positioning devices, which might be problematic in applications with very tall trees that can block GPS signals. Last, if fruit samples can be seen from both sides because of relatively open canopies, the developed framework might overestimate the number of fruit counts and thus yield load.

The second type is based on video tracking algorithms. For instance, a simple tracking algorithm has been developed for sweet pepper counting [108]. Sweet peppers were detected using a Faster RCNN model in all images. In the first image, all detections were initialized as trackers. In the rest of the images, the intersection of union (IOU) and boundary measurements (the ratio of the intersection between a tracker and a detection to the area of that detection) were used to quantify the proximity between a detection and a tracker. For a given pair of a detection and a tracker, if they had an IOU value and a boundary measurement that exceeded predetermined thresholds, the detection and tracker would be associated. When sweet peppers moved in or out of the images, the IOU and boundary measurements become problematic because of the change in the aspect ratio of the bounding boxes. To avoid this issue, start and stop zones were configured and sweet peppers detected in these zones would not be used for tracking. A small set of image sequences were used to determine the IOU and boundary measurement thresholds as well as the start and stop zones.

Although this simple tracking algorithm provided an average counting accuracy of 95.9%, it might not be stable because the thresholds could be dramatically different in various datasets. If the testing image sequences and videos are acquired in slightly different conditions, the thresholds might become invalid and result in degraded performance. As a result, the developed algorithm requires calibration for finding the proper parameters for different datasets. In addition, if fruit objects are highly occluded, the accuracy of detection-tracker association would decrease significantly. To overcome these issues, advanced video tracking algorithms (e.g., Kalman filter and optical flow) have been used to provide improved tracking performance [109, 110]. The optical flow provided motion information between two consecutive images, so the potential position of each bounding box in the current image can be estimated in the next image. Thus, the detection-tracker association was constrained by the image motion, which improved the association accuracy. The optical flow upon some assumptions, however, such as minimal motion between images and brightness consistency. The first assumption can be satisfied by controlling the data collection movement speed and image (video) acquisition frame rate, and the second assumption is relatively easy to counter. For instance, the optical flow provides degraded performance because of changes in illumination, which is unavoidable in field conditions. Also, some plant organs (e.g., flowers) are not rigid objects and are affected by wind. When the wind blows, flower shapes can change dramatically, resulting in considerable differences in pixel intensities between images.

The key concept in reconstruction-based methods is the reconstruction of a global coordinate system to which objects detected in individual images can be projected (reconstruction-based methods in Figure 4). For 2D reconstruction, global orthoimages have been reconstructed by mosaicking image sequences or video frames such that subimages of an entire crop tree or plot could be extracted from the orthoimages [111–113]. Subsequently, detection-based methods were used to detect and count plants and plant organs in the extracted subimages. In addition, a custom CNN architecture was developed to directly encode image sequences to extract both spatial and temporal features for weed detection and counting [114].

For 3D reconstruction, point clouds were obtained using either image sequences or video frames through photogrammetric algorithms (e.g., the structure from motion (SfM)) [115–118] or additional imaging sensors (e.g., LiDARs) [107, 119]. A transformation relationship was established between the 2D images and the obtained 3D point clouds, so that objects detected in 2D images could be projected to the 3D space or vice versa. As detections of the same object would significantly overlap in the 3D space, redundant detections could be eliminated to obtain accurate object quantity [112]. Additionally, 3D reconstruction-based methods enabled the extraction of additional information such as 3D location and object morphology (e.g., diameter or volume), providing great potential for comprehensive evaluation of plant/organ development.

There were several challenges, however, for the 3D reconstruction-based methods. First, significantly overlapping objects were difficult to be accurately detected, leading to inaccurate detection and counting. To overcome this issue, detection and classification-based methods have been combined. Instead of detecting individual apples, a Faster RCNN model was trained to detect apple clusters, which substantially reduced the problem complexity and improved detection accuracy. For each detected cluster, a classification-based counting method was used to determine the number of apples in that cluster. Although the combination of two strategies dramatically simplified problem complexity and improved accuracy, the developed framework was very computationally expensive. Also, individual apples could not be projected into the 3D space, which decreased the possibility of extracting additional phenotypic traits for development characterization. Another issue was the computational cost, especially the SfM technique used to obtain the 3D point clouds. The computational complexity of the SfM technique increases quadratically along with the number of the images used. While some studies attempted to use extra regulations to speed up the reconstruction process, certain environmental factors (e.g., wind) can also result in failure of 3D reconstruction using the SfM. Generally, these are ongoing issues with photogrammetric 3D reconstruction in the field, which become limiting factors for 3D reconstruction-based methods as well.

#### 3.2.3. Root System Architecture

Root phenotyping is challenging primarily because of the difficulty of imaging root system architecture (RSA) nondestructively. Most successful RSA analysis methods require researchers to dig plant root out of soil and wash them prior to imaging in an illumination-controlled environment. Therefore, segmenting roots from those images is not particularly complicated and most thresholding-based segmentation methods are sufficient. Root sample collection, however, is burdensome and could cause potential RSA damages affecting image analysis and biological interpretation. To avoid these issues, researchers try to use rhizotron systems, so RSA can be imaged using 2D imaging modalities (e.g., RGB) without human interference. As roots usually intervene with the soil, introducing several difficulties of root segmentation in images. Several studies reported the use of encode-decoder-based CNN architecture to segment roots in images [120–124]. Experimental results showed that, compared with conventional segmentation methods, CNN-based approaches generally increased the segmentation accuracy by 20% to 30% and performed more stably over images. With accurate RSA segmentations, many existing RSA analysis methods can be used to calculate important root phenotypic traits for analysis. CNN-based RSA segmentation methods also faced the challenge of limited annotated training images, so researchers tried to generate synthetic images for model training [125, 126].

While root segmentation accuracy has been improved, measuring root phenotypic traits faces another challenge in that root tips (especially second-order or smaller) can be fragmented into small pieces due to the soil occlusion. To solve this issue, a study was conducted to develop an encoder-decoder-based CNN architecture for root segmentation correction [127]. This solution considered the problem as an inpainting process that reconstructs lost connections between pieces of the same root tip. Experimental results showed that measurement accuracies of root phenotypic traits (tip length and number) using corrected segmentations increased 2 to 5 times than those using the raw segmentations. A following study further expanded the model by adding adversarial module at the patch and global levels [125]. The adversarial module helped the model to learn robust feature representations for root tip inpainting, and the two-level training helped the model to produce accurate results both locally (image patches) and globally (the whole root image). It is noteworthy that training the expanded model on a synthetic dataset led to a 72% improvement of inpainting accuracy in real root images. This would be particularly important and inspiring for many phenotyping applications that lack of annotated data for training CNNs.

Regression-based model was also developed for root tip counting to avoid extensive data annotation of RSA at the pixel level [120]. Experimental results showed that the regression-based counting method outperformed not only traditional computer vision-based counting methods but also CNN segmentation-based method. This suggests that postprocessing can be a limiting factor for the accuracy of trait measurement despite the use of CNNs for RSA segmentation.

In addition to 2D imaging, CNNs have been adopted to segment RSA in X-ray imaging [126] and to classify root tip patches in multiview 3D imaging [128], respectively. With improved segmentation and classification accuracy, a 3D structure of RSA can be extracted and analyzed, providing informative traits for biological studies. To the best of our knowledge, no study has been conducted to apply CNNs for analyzing root images collected by ground penetrating radar (GPR). Combining CNNs and GPR might be a potential way to nondestructively characterize RSA in the field.

### 3.3. Crop Postharvest Quality Assessment

While the plant phenotyping community is primarily focusing on in-season plant performance, postharvest quality is also an important focus for plant phenotyping because postharvest properties significantly affect the eventual crop productivity and quality. Based on the nature of the analysis, postharvest quality assessment can be classified into two categories: qualitative assessment and quantitative assessment. Qualitative assessment provides scores/grades for crop fruit, such as defect detection and freshness grading, whereas quantitative assessment provides continuous values for crop postharvest properties, such as firmness and soluble solid content (SSC).

Qualitative assessment of postharvest quality is similar to plant stress phenotyping, with its unique emphasis on fruit rather than plant. Most studies have investigated the use of CNNs to detect defects for fruits such as cucumbers [129], apples [130, 131], dates [132], pears [133], blueberries [134], lemons [135], and peaches [136]. These studies reported detection accuracies from 87.85% to 98.6%, which were usually 10% to 20% higher than conventional ML methods, demonstrating the advantages of using CNNs for qualitative assessment of postharvest quality. Although these efforts showed some success in addressing problems, they had several significant limitations. First, because of the availability of labeled data, most studies used very shallow CNN architecture (e.g., one convolutional layer followed by one pooling layer and one fully connected layer), meaning that the potential of CNNs for postharvest quality assessment has not been investigated fully. Even though patch-based training with data augmentation could substantially increase sample sizes, most of the image patches are highly correlated, presenting potential problems for overfitting. Second, as of writing, no studies have explored techniques for understanding the mechanism of CNNs for postharvest quality assessment, making CNN decisions and high performance unexplained. In addition to defect detection, qualitative assessment of postharvest quality includes crop grading. A CNN-based system was developed to grade the freshness of packed lettuce [137]. In this system, the CNN was trained to classify each pixel as lettuce, packaging, and artifacts using a small patch (3 by 3 pixels) surrounding that pixel. Experimental results showed that the trained CNN achieved an accuracy of 97.9% for pixel-level classification (equivalent to segmentation). The quality grading using the segmentation was comparable with grading using the images of lettuce without packing. This demonstrated the potential of using CNN to segment lettuce for grading without taking off packaging and suggests the possibility for on-shelf sorting.

Quantitative assessment of postharvest quality (e.g., sugar/acid ratio and bruising) can also be processed using CNNs. A study was conducted to develop a CNN-based regression model for estimating sugar/acid ratio for citrus [138]. An excitation-emission matrix (EEM) is an image of a measuring sample in which the *x*-axis indicates excitation wavelengths (nm), the *y*-axis indicates emission wavelengths (nm), and the intensity of a pixel (*x* and *y*) is sample fluorescence excited at *x* (nm) by using emission light at *y* (nm). Images of EEM were used as input to train a custom CNN with 8 layers for regression. Sugar/acid ratio values were estimated using the trained CNN models for 20 testing samples, and results showed that the CNN-based regression model achieved the lowest prediction error of 2.48, which was 2 to 3 times less than conventional regression models. Another study investigated the use of a fully convolutional network (FCN) for segmenting bruised, nonbruised, and calyx end tissues for blueberries [139]. The FCN model was based on a VGG-16 network. Experimental results showed that the developed approach provided segmentation accuracies of 73.4% to 81.2%, which were substantially higher than the SVM-based segmentation method (46.6%). A partial reason for this is that the spectra of the calyx end were similar to bruised tissues, and thus, using conventional classifiers was difficult to accurately differentiate them. In contrast, CNN-based approaches can learn other features, such as shape and position, which significantly contribute to the improvement of the segmentation accuracy of the calyx end. This study was the only case using an end-to-end CNN model for postharvest quality assessment and provided valuable results for future studies. However, there were several issues in the study. First, hyperspectral images have many more channels than RGB images, which leads to an issue with using transfer learning. In this study, an additional layer was developed to reduce the dimensionality of raw hyperspectral images from an arbitrary value to 3, so that weights pretrained on other datasets could be used for the bruising dataset. However, experimental results showed that the FCN models trained using transfer learning were less accurate than those trained entirely using the new dataset. The authors stated that this was primarily because of the difference between the bruising dataset and the ImageNet dataset, meaning that the majority of learned filters from the ImageNet are not useful for bruising detection. This poses a critical question of whether publicly available datasets can benefit postharvest quality assessment studies that rely more heavily on advanced imaging modalities (e.g., multispectral and hyperspectral imaging) than on RGB imaging.

All the methods reviewed for various phenotyping applications are summarized in Table 2. Therefore, readers could quickly identify potential solutions to problems similar in their applications.

## 4. Discussion

### 4.1. Data Availability

The availability of diverse annotated datasets is a key factor for all DL-related studies. Adequate annotated datasets enable and ensure the swift development and evolution of DL methods. This generally holds true for domain applications such as plant phenotyping. For biotic/abiotic stress phenotyping, data annotation is relatively straightforward and has resulted in several large publicly available datasets, such as PlantVillage. For plant development, as sensing technologies are under development, few datasets are publicly available and there are also few annotated datasets. As the main purpose of DL is to learn features from data, it is very difficult to develop (or even use) DL techniques without sufficient annotated data. Data annotation itself comes with several challenges for plant phenotyping. First, data annotation sometimes requires domain expertise. For instance, it is easy to label cars, whereas it is difficult to label particular plant diseases because of the need of domain knowledge and working experiences. Thus, it is not easy to crowdsource annotation tasks, which limits the efficiency and throughput of data labeling. Second, unlike common uses, plant phenotyping oftentimes relies on advanced imaging techniques, such as thermal and hyperspectral imaging. Labeling of those data is considerably more difficult than labeling color images because of fewer visual cues. Third, there are many phenotyping applications that require object detection and segmentation (semantic, instance, or panoptic), and these applications require instance-level (bounding boxes) and pixel-level (masks) annotations. Those are very time-consuming tasks and become the major limiting factor for using DL in plant phenotyping. Some challenges are common for general computer vision tasks, and researchers have proposed and developed some solutions. To significantly reduce the requirements of labeled data, one of the most important techniques is transfer learning. Transfer learning relies on the assumption that a very large dataset ensures that the learned filters are common for other datasets. Thus, for some domain applications with limited labeled data, transfer learning could significantly improve training efficiency and accuracy. However, the key challenge is whether phenotyping datasets are similar to very large common datasets (e.g., ImageNet or MS COCO), especially for some phenotyping applications using advanced imaging techniques (e.g., hyperspectral imaging). Active learning is another effort for the reduction in the cost of data labeling. Compared with conventional data annotation, active learning is aimed at finding and labeling samples that maximize model performance. Thus, the majority of samples do not need to be annotated to save time and labor cost. Crowdsourcing is also a viable way for data annotation, which requires less investment in labor cost. Some studies have demonstrated the capability of using crowdsourcing for quickly labeling large image datasets for machine learning applications. In particular, there are some commercial services for crowdsourcing annotation such as Amazon Mechanical Turk and CrowdFlower. Through those services, a reasonable quality and throughput can be ensured for data annotation. In addition, GANs have been originally proposed as a generative module for deep learning [142], but they are very promising tools in combination with CNNs to solve computer vision tasks [143]. In particular, GANs can be used to generate synthetic data to increase the data availability and enhance the data diversity. As the technical community is making significant improvements of GAN architecture and training, the phenotyping community could better adopt them to solve the data availability issue in plant image analysis.

### 4.2. Adoption of DL Methods for Plant Phenotyping

Another important consideration is the adoption of DL methods for plant phenotyping. Technology companies have released various DL frameworks that accelerate the development and implementation of new DL algorithms such as reinforcement learning and attention mechanism. In particular, the DL community encourages researchers to share source codes of original studies to facilitate other research projects. These efforts considerably ease the adoption of the latest DL methods for domain applications, such as plant phenotyping. However, there is still a delay in the use of the latest technologies for plant phenotyping. This likely occurs for three reasons. First, some of the latest DL methods require a significant investment in computational power, which cannot be achieved easily in ordinary research labs. Second, original DL solutions might not be directly usable for plant phenotyping applications. Additional efforts are necessary to adopt these advanced DL solutions, and sometimes these efforts are technically challenging. Educational programs are expected to be promoted, so more domain experts (e.g., agricultural engineers and plant scientists) can gain adequate knowledge and skills to expedite the adoption/modification of DL methods for challenging agricultural and biological applications. Thirdly, large private companies, who invest heavily in plant phenotyping, do not disclose their research efforts in this area to the public.

### 4.3. CNNs for 3D Image Processing

3D imaging, an important imaging technique, has not been mentioned yet. An important plant phenotyping task is to characterize and understand plant morphology. While few studies reported the use of CNN in a scenario with 3D imaging, they have primarily focused on the detection in 2D images and projected the detections in 3D for processing, such as removal of redundant detections and determinations of detections with occlusions. None of them really utilized CNNs for plant morphology characterization and understanding. In particular, 3D point clouds can be collected using various approaches (such as LiDAR and photogrammetric methods) in plant phenotyping applications, and most of them need to be analyzed using conventional 3D processing methods. One possible reason is that even the DL community has not delivered many reliable tools for 3D point cloud processing. PointNet and PointNet++ are pioneering work for processing 3D point clouds, but they are limited to the number of points in each model (a couple of thousand points). If the point cloud is too large, there is no efficient computational solution for network training and inference. Thus, much 3D imaging work requires technical development from the DL community.

## 5. Conclusions and Future Directions

In this review, CNN-based solutions to image-based plant phenotyping were comprehensively reviewed to provide advantages and disadvantages of using them for different tasks of plant phenotyping. Through these studies, CNN-based solutions demonstrated their great potential for solving the most challenging problems encountered in various plant phenotyping applications. In particular, some types of end-to-end CNN architecture have streamlined the process of extracting phenotypic traits from images significantly. This would enable the improvement of data processing and ultimately plant phenotyping applications.

Several future research directions that use CNNs for plant phenotyping are identified. The first direction is to enrich the availability of labeled data. Although there are some datasets publicly available (e.g., ImageNet and MS COCO), they are not well integrated and designed for agricultural applications. This holds true especially for postharvest quality assessment that utilizes different imaging modalities and has limited samples. The second direction is to customize a deep learning framework that can facilitate the adoption of the latest DL techniques for plant phenotyping applications. Such a framework could provide a common interface for algorithm integration, so that newly developed models and tools can be added for use with little or no development effort, such as visualization tools for model explanation and reinforcement learning for model improvement. Experiences of adopting these newly developed DL methods can be promoted through educational programs and training workshops to advance DL-based data analytics for agricultural applications. The third direction is to adopt and develop CNN architecture for direct 3D and multimodal data processing, especially skeleton extraction, branch-pattern classification, and plant-development understanding.

## Figures and Tables

**Table 1:** Summary of major CNN architecture developed for image classification, object detection, and semantic and instance segmentation.

Model	Vision task	Key concept	Source code (third-party implementation)
AlexNet	Image classification	A five-layer CNN architecture	https://github.com/TensorFlow/models/blob/master/research/slim/nets/alexnet.py (TensorFlow) https://github.com/pytorch/vision/blob/master/torchvision/models/alexnet.py (PyTorch)

ZFNet	Image classification	Feature visualization for model improvement	https://github.com/caffe2/models/tree/master/zfnet512 (Caffe2)

VGGNet	Image classification	Small-sized (3 by 3) convolutional filters to increase the depth of CNNs (up to 19 layers)	http://www.robots.ox.ac.uk/~vgg/research/very_deep/ (Caffe)^∗^

Inception family	Image classification	Inception modules for increasing the width of CNNs and therefore the capability of feature representation	https://github.com/TensorFlow/models/tree/master/research/inception (TensorFlow) https://github.com/pytorch/vision/blob/master/torchvision/models/inception.py (PyTorch)

ResNet family	Image classification	Residual representation and skip connection scheme to enable the training of very deep CNNs (up to 1000 layers)	https://github.com/TensorFlow/models/tree/master/official/resnet (TensorFlow) https://github.com/pytorch/vision/blob/master/torchvision/models/resnet.py (PyTorch)

DenseNet	Image classification	Dense block modules to substantially decrease the number of model parameters (therefore computational cost) and strengthen feature propagation (therefore feature learning capability)	https://github.com/liuzhuang13/DenseNet (supports multiple DL framework)^∗^

NASNet	Image classification	Reinforcement learning on a small dataset to find optimal convolutional cells that are used to build a CNN architecture for a large dataset	https://github.com/TensorFlow/models/tree/master/research/slim/nets/nasnet (TensorFlow)

RCNN family	Object detection	A two-stage framework to generate regions of interest (ROIs) and then predict the class label and calculate the bounding box coordinates for each ROI	https://github.com/TensorFlow/models/tree/master/research/object_detection (TensorFlow) for Faster RCNN https://github.com/facebookresearch/Detectron (Caffe2) for R-FCN, and Fast/Faster RCNN

YOLO family	Object detection	A one-stage framework to regress both class labels and bounding box coordinates for each grid cell on the last feature map	https://pjreddie.com/darknet/yolo/ (C++)^∗^

SSD	Object detection	A one-stage framework to regress class labels and bounding box coordinates for anchors in each grid cell on feature maps extracted from different convolution layers (thus different resolutions)	https://github.com/weiliu89/caffe/tree/ssd (Caffe)^∗^ https://github.com/TensorFlow/models/tree/master/research/object_detection (TensorFlow) for SSD

RetinaNet	Object detection	A one-stage framework to use focal loss that is a new loss function to solve the foreground-background class imbalance problem	https://github.com/facebookresearch/Detectron (Caffe2) for RetinaNet^∗^

FCN	Semantic segmentation	Fully convolutional architecture to train and predict classes at the pixel level in an end-to-end manner for semantic segmentation	https://github.com/shelhamer/fcn.berkeleyvision.org (Caffe)^∗^ https://github.com/shekkizh/FCN.TensorFlow (TensorFlow) https://github.com/wkentaro/pytorch-fcn (PyTorch)

U-Net	Semantic segmentation	An encoder-decoder architecture for semantic segmentation	https://lmb.informatik.uni-freiburg.de/people/ronneber/u-net/ (Caffe)^∗^ https://github.com/jakeret/tf_unet (TensorFlow) https://github.com/milesial/Pytorch-UNet (PyTorch)

DeepLab family	Semantic segmentation	Atrous convolution operation to simultaneously increase receptive field and reduce the computation complexity to improve the segmentation accuracy; fully connected conditional random field (CRF) as a postprocessing method to improve the segmentation accuracy	https://bitbucket.org/aquariusjay/deeplab-public-ver2/src/master/ (Caffe)^∗^ https://github.com/TensorFlow/models/tree/master/research/deeplab (TensorFlow) https://github.com/jfzhang95/pytorch-deeplab-xception (PyTorch)

Mask RCNN	Instance segmentation	Masking head with ROI align operation on top of the Faster RCNN model to significantly improve segmentation accuracy	https://github.com/facebookresearch/Detectron (Caffe2)^∗^ https://github.com/tensorflow/models/tree/master/research/object_detection (TensorFlow) for Mask RCNN

Note: ^∗^source code provided by original authors.

**Table 2:** Summary of CNN-based data analysis approach in imaging-based plant phenotyping.

Phenotyping category	Phenotyping task	Main processing approach	Particular improvement strategy	References
Plant stress	Stress detection and classification	Image classification	NA	[43, 44, 47–53]
			Sliding window	[45, 57]
			Explainable visualization	[55, 56]
			Advanced imaging	[58]
			Synthetic data augmentation	[54]
		Object detection	NA	[46]

Plant development	Plant lodging	Image classification	NA	[68]
	Canopy morphology measurement	Object detection	NA	[61, 65]
		Semantic segmentation	NA	[59, 63, 64]
	Leaf morphology measurement	Instance segmentation	NA	[60, 62]
	Characterization of plant growth pattern	Combination of CNN and other DL methods	NA	[66, 67]

Plant development	Counting plant/plant organs in still images	Regression	NA	[69, 70, 72, 79]
			Synthetic data augmentation	[71, 73]
			Multiscale and multimodal data fusion	[74, 75]
			Nonsupervised learning mode	[76, 78, 80]
			Explainable visualization	[77]
		Image classification	NA	[82, 83]
		Object detection	NA	[84–91, 93, 94]
			Sliding window	[92, 95]
			Synthetic data augmentation	[95]
		Semantic segmentation	NA	[96–101]
			Sliding window	[101]
		Instance segmentation	NA	[102–106]
			Synthetic data augmentation	[102, 103, 105, 106]

Plant development	Counting plant/plant organs in image sequences and videos	Object detection	2D orthoimage reconstruction	[111–113]
			3D structure reconstruction	[107, 115–119]
			Video tracking	[108–110]
		Semantic segmentation	Movement encoding	[114]

Plant development	Counting root tips	Regression	NA	[120]
	Root system architecture segmentation	Semantic segmentation	NA	[120–124]
			Inpainting for oversegmentation correction	[125, 127]
			Advanced imaging	[126, 128]
			Synthetic data augmentation	[125, 126]

Postharvest quality	Fruit chemical composition measurement	Regression	NA	[138]
	Fruit defect detection	Image classification	NA	[131, 132, 135, 136, 140]
			Advanced imaging	[134]
			Sliding window	[129, 137]
	Fruit defect quantification	Semantic segmentation	NA	[141]
			Advanced imaging	[139]
